# A Description of Risk Factors for Non-alcoholic Fatty Liver Disease in the Southern Community Cohort Study: A Nested Case-Control Study

**DOI:** 10.3389/fnut.2020.00071

**Published:** 2020-05-21

**Authors:** Sudipa Sarkar, Loren Lipworth, Edmond K. Kabagambe, Aihua Bian, Thomas G. Stewart, William J. Blot, T. Alp Ikizler, Adriana M. Hung

**Affiliations:** ^1^Division of Endocrinology, Diabetes and Metabolism, Department of Medicine, Johns Hopkins University School of Medicine, Baltimore, MD, United States; ^2^Division of Epidemiology, Vanderbilt University Medical Center, Nashville, TN, United States; ^3^Vanderbilt Center for Kidney Disease, Vanderbilt University Medical Center, Nashville, TN, United States; ^4^Department of Biostatistics, Vanderbilt University Medical Center, Nashville, TN, United States; ^5^International Epidemiology Institute, Rockville, MD, United States; ^6^Division of Nephrology and Hypertension, Department of Medicine, Vanderbilt University Medical Center, Nashville, TN, United States

**Keywords:** low socioeconomic status, micronutrients, macronutrients, ethnicity, non-alcoholic fatty liver disease

## Abstract

**Background:** Non-alcoholic fatty liver disease (NAFLD) is associated with obesity and hypercholesterolemia. In addition, total fat and folate intake have been associated with NAFLD.

**Aims:** We investigated risk factors for NAFLD among individuals of largely low socioeconomic status, and whether these associations differed by race.

**Methods:** A nested case-control study was conducted within the Southern Community Cohort Study. Through linkage of the cohort with Centers for Medicare and Medicaid Services, International Classification of Diseases, Ninth Revision, Clinical Modification codes were used to identify incident NAFLD cases. Controls were matched 4:1 to cases on enrollment age, sex, and race. A logistic regression was used to estimate odds ratios for the associations of NAFLD with covariates of interest.

**Results:** Neither total fat nor folate intake was significantly associated with NAFLD. Hypercholesterolemia (odds ratio 1.21) and body mass index (75th vs. 25th percentile) for blacks (odds ratio 1.96) and whites (odds ratio 2.33) were associated with an increased risk of non-alcoholic fatty liver disease. No significant interaction with race for any of the studied variables was noted.

**Conclusions:** Both hypercholesterolemia and increasing body mass index, but not total fat and folate intake, were risk factors for NAFLD in the Southern Community Cohort Study.

## Introduction

The prevalence of non-alcoholic fatty liver disease (NAFLD) is generally estimated to be 20–30% in Western countries (1). Because of its high prevalence and associated adverse health effects, NAFLD has been identified as a significant contributor to increased health care costs, even after controlling for factors such as age, body mass index (BMI), diabetes, and other comorbidities (2). Furthermore, about 10–22% of patients with NAFLD develop non-alcoholic steatohepatitis (NASH) (3), a progressive form of NAFLD (4), and by 2020, NASH is projected to be the primary etiology for liver transplantation (5). Other than lifestyle changes including decreased caloric intake or increased physical activity, there are no currently recommended long-term treatments for NAFLD (6).

Variations in the prevalence of NAFLD across ethnic groups have been noted (7), and NAFLD is more prevalent in countries with higher per capita gross national incomes (8). One explanation for racial disparities in NAFLD is thought to be differences in adipose tissue distribution, specifically visceral adiposity (7, 9, 10). With respect to other risk factors for NAFLD development, there is emerging interest in the role of nutrients such as dietary folate and fatty acids. Data from animal models in which there is folate restriction or supplementation in high risk mice reveal a significant association with liver injury or NAFLD, and human studies, while less consistent, suggest a positive association between low folate and/or vitamin B12 (and high homocysteine) and NAFLD (11–19). Increased total fat intake has been found to be positively associated with prevalent NASH (6, 20). Of note, lower folate and other micronutrient intakes have been reported in minority populations (21, 22), but there are limited data on risk factors for NAFLD among low-income populations, who tend to have less access to nutrient-rich diets and to consume energy-dense diets (23). Furthermore, it is not known whether there exists an interaction between race and specific dietary factors that may influence the risk of NAFLD.

The Southern Community Cohort Study (SCCS) is a large ongoing prospective study of adult participants, two-thirds of whom are black and over half of whom have an annual household income below $15,000 (24, 25). This cohort presents an ideal context in which to study the joint relationship between race and dietary factors, such as total fat and folate intake, in NAFLD. In this case-control study nested within the prospective SCCS, we sought to identify factors contributing to incident NAFLD in the SCCS, such as diabetes, hypercholesterolemia, BMI, and lifestyle factors such as daily energy expenditure, and to determine whether there are differences by race in the effect of BMI or dietary factors (i.e., intake of total fat and folate) on NAFLD.

## Materials and Methods

### Cohort Description

The sample for this nested case-control study was derived from individuals enrolled in the SCCS. Between March 2002 and September 2009, ~85,000 participants age 40–79 years and resident in 12 states in the southeastern United States, were enrolled in the SCCS. Approximately 86% of participants were recruited from community health centers (CHCs), settings that provide primary health and preventive care to underserved populations (25), while the remaining 14% were recruited from the general population. At cohort enrollment, CHC participants completed a computer-assisted personal interview, and general population participants completed and mailed in the study questionnaires. Detailed descriptions of SCCS methods have been previously published (25). Informed consent was obtained from all participants, and the study protocols were approved by the Institutional Review Boards at both Vanderbilt University Medical Center and Meharry Medical College.

### Study Population

The SCCS cohort was linked to the Centers for Medicare and Medicaid Services (CMS) Research Identifiable Files from January 1, 1999 to December 31, 2010 using Social Security number, date of birth, and first and last name in order to ascertain claims for medical conditions that were diagnosed after study enrollment (25, 26). The eligible source population for this study was selected from the SCCS using the following inclusion criteria: age ≥ 65 years old at the time of enrollment in the SCCS, or age < 65 years old at SCCS enrollment and having ≥ 2 CMS claims between enrollment and the end of follow-up (December 31, 2010). These criteria were used in order to increase the likelihood of continuous Medicare and/or Medicaid coverage from enrollment to the end of the follow-up period in order for NAFLD cases to be ascertained.

Based on a set of International Classification of Diseases, Ninth Revision, Clinical Modification (ICD-9-CM) codes previously used to identify individuals with NAFLD in the Surveillance, Epidemiology, and End Results (SEER) registries (27), participants in the source population who fulfilled the criteria for NAFLD after the date of SCCS enrollment were identified as incident NAFLD cases. Participants who had NAFLD before enrollment in the SCCS were considered to have prevalent NAFLD and excluded from the eligible study population.

**Figure 1:**
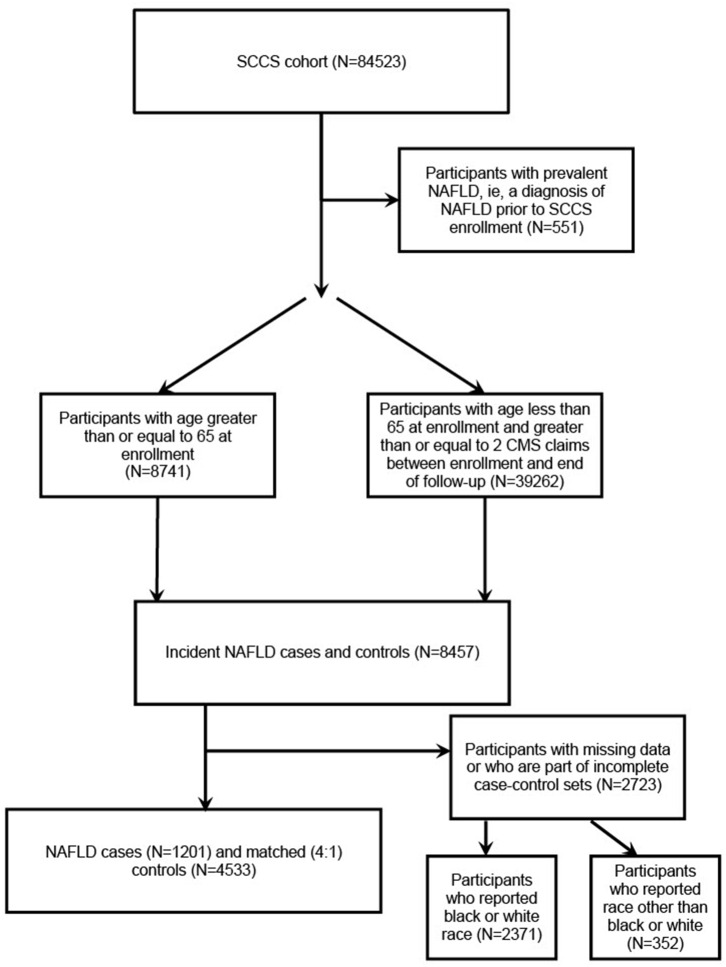
Flowchart of selecting cases and controls from the SCCS cohort.

The inclusion criteria for ICD-9-CM codes were as follows: 571.5 (cirrhosis of liver without alcohol), 571.8 (other chronic nonalcoholic liver disease), or 571.9 (unspecified chronic liver disease without alcohol). The exclusion criteria included having ≥ 1 of the following ICD-9-CM codes: 070.41, 070.44, 070.51, 070.7, V02.62 (hepatitis C virus (HCV) infection), 070.2, 070.3, 070.42, 070.52, V02.61 (hepatitis B virus (HBV) infection), 571.0 (alcoholic liver disease), 571.1 (alcoholic hepatitis), 571.2 (alcoholic cirrhosis of the liver), 571.3 (alcoholic liver damage), 571.4 (autoimmune hepatitis), 571.6 (biliary cirrhosis), 275.0 (hemochromatosis), 275.1 (Wilson's disease), 303, 305, V11.3, V79.1, 291 (other alcohol-related disorders), and 042 (human immunodeficiency virus (HIV) disease). NAFLD cases, as well as controls, who reported having hepatitis on the baseline questionnaire were excluded. Because the number of participants who reported a race other than black or white was small (about 5%), they were removed from the analyses. Individually matched controls were randomly selected by incidence density sampling from among cohort members of similar age at SCCS enrollment (± 5 years), sex, and race. Controls were defined as participants from the eligible population who did not fulfill the ICD-9-CM definition for diagnosis of incident NAFLD and were individually matched 4:1 to cases (Figure 1). As such, 1,201 cases and 4,533 matched controls were identified.

### Data Collection

The baseline questionnaire (available at http://www.southerncommunitystudy.org/) collected information on participants' demographic characteristics, personal and family medical history, lifestyle and anthropometric factors. Dietary intake was assessed at baseline using an 89-item food-frequency questionnaire (28). Participants selected the amount of average intake over the past year for different food items and supplements, with a range from “Never” to “2+/day.” Intake levels for individual food items were derived from race- and sex-specific portion size information from the National Health and Nutrition Examination Study and the United States Department of Agriculture (USDA) Continuing Survey of Food Intakes by Individuals. Total energy and nutrient intake were derived from these data (23). Calculation of folate intake took into account intake from food sources only.

Daily energy expenditure was derived from the participants' reports of how much daily light, moderate, and strenuous activity they performed and expressed as standard metabolic equivalent-hours per day (MET-h/day), which indicates the intensity and duration of activity. These calculations were made using standard methods described in the Compendium of Physical Activity, which describes the energy cost of more than 600 different activity-related behaviors (29).

### Statistical Analyses

The design of the study was a nested case-control study. The data were modeled using a conditional logistic regression model, conditioning on the matched case-control sets (each set included four controls and one case), with NAFLD as the dependent variable. Participants were excluded from the analysis if they were part of incomplete case-control sets or were missing any of the following variables: age, race, sex, history of diabetes, hypercholesterolemia, myocardial infarction (MI) or coronary artery bypass surgery, BMI, annual household income (< $15,000, ≥ $15,000), number of daily alcoholic drinks, daily energy expenditure, and last visit to a doctor (prior to enrollment visit, in months) (Supplementary Table 1). The independent variables, all self-reported on the baseline questionnaire, were included in a single model and were as follows: BMI, history of physician diagnosis of diabetes (no or yes), hypercholesterolemia (no or yes), MI or coronary artery bypass surgery (no or yes), annual household income (< $15,000, ≥ $15,000), number of daily alcoholic drinks, daily energy expenditure, and last visit to a doctor (prior to enrollment visit, in months). BMI was calculated from height and weight, which were reported by the participants. Folate and total fat intake, the primary dietary variables of interest, were derived from a validated food frequency questionnaire and adjusted for total daily energy intake using the residual method (30, 31). BMI, daily energy expenditure, number of daily alcoholic drinks, last visit to a doctor, daily energy intake, total fat intake, and folate intake were added in the model as non-linear variables using restricted cubic splines. Next, we tested for interactions between race and our primary dietary variables (folate and total fat) as well as between race and BMI in the conditional logistic regression model with the matching variables and covariates specified above. The analyses were performed using R version 3.3.0 (2016-05-03). *P-*values ≤ 0.05 were considered statistically significant.

## Results

Of the 84,523 participants of the SCCS, 551 participants with a diagnosis of NAFLD prior to SCCS enrollment were not included in our study. Of the remaining participants, 8,741 participants had an age ≥ 65 years at enrollment and 39,262 participants had an age < 65 years but had ≥ 2 CMS claims between enrollment and the end of follow-up. Of these, 8,457 participants were identified as incident NAFLD cases or controls, but 2,723 participants were excluded for having missing data or being part of an incomplete case-control set (Figure 1).

Baseline characteristics of the 1,201 cases and 4,533 matched controls are presented in Table 1. As a result of matching, the median age at enrollment of both the cases and controls was 58 years old [interquartile range (IQR) 50–64]. Fifty-seven percentage of the cases and 57% of the controls were black, and 23% were male. Of the cases, 37% had diabetes and 52% had hypercholesterolemia, while among the controls, 29% had diabetes and 45% had hypercholesterolemia.

**Table 1:** Baseline characteristics of participants in the Southern Community Cohort Study.

Characteristic^*^	**Cases (*n* = 1,201)**	**Controls (*n* = 4,533)**
Age (years)	58 (50–64)	58 (50–64)
Race		
Black	679 (56%)	2,583 (57%)
White	522 (43%)	1,950 (43%)
Sex		
Female	920 (77%)	3,513 (77%)
Male	281 (23%)	1,020 (23%)
History of diabetes	443 (37%)	1,299 (29%)
History of hypercholesterolemia	627 (52%)	2,019 (45%)
History of MI or bypass	163 (12%)	472 (10%)
BMI (kg/m^2^)	33 (28–39)	30 (26–36)
Household income < $15,000/year	715 (60%)	2,758 (61%)
Number of alcoholic drinks/day	0.00 (0.00–0.02)	0.00 (0.00–0.15)
Current alcohol use	375 (31%)	1,907 (42%)
Daily energy expenditure (MET-h/day)	12.6 (6.6–21.8)	13.3 (7.0–23.0)
Last visit to a doctor (prior to enrollment, in months)	1 (0–2)	1 (0–3)
Daily energy intake (kcal/day)	1,856 (1,333–2,649)	1,956 (1,395–2,754)
Total daily fat intake (g/day)	70 (49–103)	73 (51–107)
Total daily folate intake (μg/day)	411 (287–594)	422 (298–597)

* *Values are number of participants (percent) for categorical characteristics or median (IQR) for continuous characteristics*.

*MI, myocardial infarction; BMI, body mass index; MET, metabolic equivalent-hours*.

As shown in Table 2, total fat intake (75th percentile vs. 25th percentile of intake) was not associated with odds of being diagnosed with NAFLD among either blacks (odds ratio (OR) = 0.98; 95% confidence interval (CI) 0.73–1.32) or whites (OR = 0.99; 95% CI 0.72–1.35), nor was there evidence of interaction with race (*p* > 0.10). Folate intake (comparing the 75th percentile to the 25th percentile of intake) was associated with a non-significant decrease in odds of being diagnosed with NAFLD (OR = 0.83; 95% CI 0.60–1.15) for whites, but not for blacks (OR = 1.10, 95% CI 0.82–1.47), but there was no statistically significant interaction (*p* > 0.10).

**Table 2:** Odds ratios (95% confidence intervals) for non-alcoholic fatty liver disease.

Variable^*^	**Odds ratio**	**95% confidence interval**
BMI (kg/m^2^)		
Black (75th percentile vs. 25th percentile)	1.96	1.51–2.56
White (75th percentile vs. 25th percentile)	2.33	1.70–3.19
Hypercholesterolemia	1.21	1.05–1.40
MI or coronary artery bypass surgery	0.96	0.78–1.20
Diabetes	1.10	0.94–1.28
Number of daily alcoholic drinks (75th percentile vs. 25th percentile)	0.95	0.93–0.97
Daily energy expenditure (MET-h/day) 75th percentile vs. 25th percentile	0.95	0.80–1.12
Last visit to a doctor (prior to enrollment, in months) 75th percentile vs. 25th percentile	0.88	0.75–1.03
Household income < $15,000/year	0.87	0.75–1.01
Daily energy intake (kcal/day) 75th percentile vs. 25th percentile	0.91	0.80–1.03
Total fat intake (g/day)		
Black (75th percentile vs. 25th percentile)	0.98	0.73–1.32
White (75th percentile vs. 25th percentile)	0.99	0.72–1.35
Folate intake (μg/day)		
Black (75th percentile vs. 25th percentile)	1.10	0.82–1.47
White (75th percentile vs. 25th percentile)	0.83	0.60–1.15

* *All variables were entered into the model simultaneously*.

*BMI, body mass index; MI, myocardial infarction; MET, metabolic equivalent-hours*.

**Figure 2:**
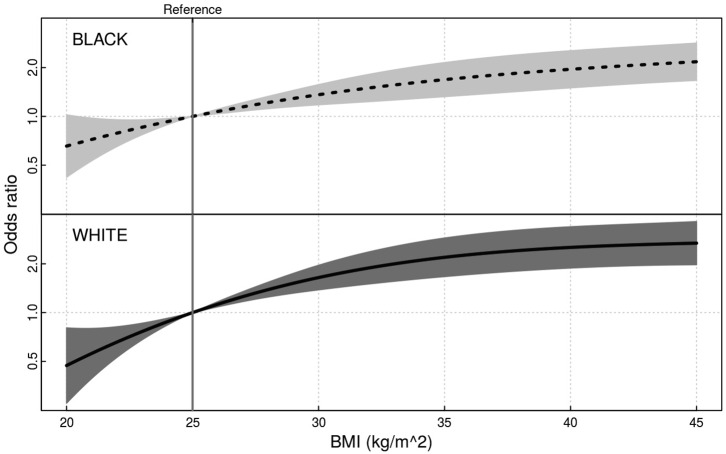
Odds ratio of NAFLD over BMI by race.

Having a higher BMI (75th percentile compared to 25th percentile) was associated with significantly greater odds of being diagnosed with NAFLD for both blacks (OR 1.96; 95% 1.51–2.56) and whites (OR 2.33; 95% CI 1.70–3.19), but the interaction of race and BMI was not significant (*p* > 0.10) (Figure 2). There was an inverse association of small magnitude with the number of daily alcoholic drinks (75th percentile of intake vs. 25th percentile of intake) and NAFLD (OR = 0.95; 95% CI 0.93–0.97). Hypercholesterolemia was associated with significantly greater odds of being diagnosed with NAFLD (OR = 1.21; 95% CI 1.05–1.40). Diabetes, MI or coronary artery bypass surgery, time since last doctor's visit, annual income, daily energy expenditure, and total energy intake were not significantly associated with NAFLD in the model (Table 2).

## Discussion

In this study of black and white middle aged and older adults of generally low income who were eligible for Medicaid or Medicare in the SCCS, we found that BMI and hypercholesterolemia were associated with greater odds of NAFLD. We also showed that folate intake and total fat intake were not significantly associated with NAFLD, and that neither of these associations, nor that of BMI with NAFLD, varied by race.

Folate intake varies by race/ethnicity and by SES in the United States. For example, Black and Hispanic women have lower pre-pregnancy red blood cell folate concentrations than Non-Hispanic White women (32), despite mandatory folic acid fortification in the United States. In addition, in the lowest quartile income groups compared to the highest quartile income group, decreases were observed in the absolute differences in red blood cell folate concentration after mandatory folic acid fortification, but increases in the relative ratios were noted (33).

Both decreased folate intake and increased total fat intake have been directly associated with prevalent NAFLD or NASH in previous studies (19, 20). Among obese female patients undergoing bariatric surgery, Hirsch et al. found that serum folate levels were greater in those with normal liver biopsies [27.7 ± 7.04 nmol/L, (mean ± SD)] than that of those with NAFLD (21.1 ± 7.9 nmol/L, *p* = 0.005) (19). On a similar note, among obese female children, Frelut et al. found that elevated alanine aminotransferase levels, an indicator of NAFLD, were noted in those who were homozygous for a mutation in folate metabolism compared to those who were not homozygous for the mutation (14). Moreover, in a study from South Korea, folate intake was associated with a decreased risk of NAFLD (OR = 3.37) in male participants (15), while another study from Iran showed that the mean intake of folate in healthy controls was significantly higher than that of patients with NAFLD (18). With regards to fat intake, in a cross-sectional study of NAFLD participants, Vilar et al. showed that total lipid intake as a percent of total energy intake among participants with NASH was greater at 37.5 ± 8.0% (mean ± SD) than that of participants with steatosis alone at 31.2 ± 7.8% (*p* = 0.003) (20). While the Vilar et al. study compared participants with biopsy-proven NASH vs. simple hepatic steatosis (20), our study compared those with and without NAFLD, based on ICD-9-CM codes.

We found that both hypercholesterolemia and higher BMI were associated with an increased risk of NAFLD and that the association between BMI and NAFLD was not significantly modified by race. Hypercholesterolemia is a known risk factor for NAFLD (34), and other traditional risk factors for NAFLD, such as diabetes and obesity, disproportionately affect black adults (35, 36), who comprise a substantial proportion of the SCCS. However, the distribution of body fat, specifically percent visceral fat, is thought to be a particular factor that accounts for racial differences in the prevalence of NAFLD (10), and BMI does not reflect body fat distribution or distinguish between adipose tissue, fat free mass and skeletal mass which vary widely across multi-ethnic populations for a given BMI value (37).

In the current study, the number of daily alcoholic drinks had a small inverse relationship with NAFLD. This finding likely arises because NAFLD is usually diagnosed after excluding other causes of liver disease, such as chronic alcohol use, and participants who reported significant alcohol use would be less likely to be diagnosed as having NAFLD. In this study, current drinkers, defined as those participants who had ≥ 1 daily alcoholic drinks, reported mild to moderate alcohol consumption, with medians of 1.3 daily alcoholic drinks among controls and 1.1 among cases. Dunn et al. found that among patients with biopsy-proven NAFLD, those who reported moderate alcohol consumption had decreased odds for progressive liver disease with fibrosis (OR = 0.56, 95% CI 0.41–0.77) than non-drinkers (38). As such, the findings of our study are consistent with the Dunn et al. (38) study, but since heavy alcohol intake may preclude the diagnosis of NAFLD, it is difficult to draw any conclusions on alcohol use and NAFLD.

NAFLD has become increasingly widespread, but it has been relatively less studied among low socioeconomic status (SES) individuals. Zhu et al. (8) noted that in countries in Asia and Europe with a higher national income, there were higher prevalences of NAFLD. The authors postulated that this was the result of more sedentary behavior and a move away from traditional diets toward diets with an increased amount of calories (8). In the SCCS population of generally low SES, the observed lower risk of NAFLD among those with lower income, although not statistically significant, is suggestive of a similar association with income. However, in the current study, we accounted for total energy intake in the model.

The strengths of the study include studying NAFLD in the setting of a large, low SES cohort, with a majority of black participants. This represents a population that has previously been less studied with respect to NAFLD, and one with a relatively high burden of risk factors previously shown to be associated with NAFLD. Moreover, the use of a validated dietary questionnaire in the SCCS allowed examination of associations between nutrient intake, specifically total fat and folate, and NAFLD.

The current study has some limitations. NAFLD was identified through CMS claims using a set of ICD-9-CM codes. Clinically, methods of identifying NAFLD include using liver aminotransferase levels and hepatic imaging. However, like us, other groups have used ICD-9-CM codes to determine which patients had NAFLD, as in SEER registries, which were linked to Medicare data (27). Although the use of ICD-9-CM codes provides a means by which to determine which individuals have NAFLD in a large cohort such as the SCCS, it likely underestimates the true number of individuals with NAFLD because NAFLD is underdiagnosed by clinicians (39). Future directions include determining the sensitivity and specificity of ICD-9-CM code algorithms for identifying NAFLD in large population cohorts, as well as using natural language processing and other bioinformatics approaches in electronic health records to identify NAFLD. Another limitation is the use of a case-control study design. However, we did use interaction terms between race and BMI, folate, and total fat, respectively, to help mitigate bias.

Our results suggest no significant associations between total fat intake and folate intake with NAFLD in this predominantly black, low SES cohort. Hypercholesterolemia and BMI, generally accepted as traditional NAFLD risk factors, were associated with an increased risk of NAFLD among both blacks and whites >40 years old.

## Data Availability Statement

The datasets generated for this study will not be made publicly available. Requests for data need to be reviewed and approved by the SCCS Data and Biospecimen Use Committee. Requests can be made directly to the Southern Community Cohort Study: https://www.southerncommunitystudy.org/.

## Ethics Statement

The studies involving human participants were reviewed and approved by Institutional Review Boards at Vanderbilt University Medical Center and Meharry Medical College. The patients/participants provided their written informed consent to participate in this study.

## Author Contributions

SS, LL, EK, and AH participated in designing the study and helped draft the manuscript. SS, LL, EK, AB, TS, and AH analyzed and interpreted the data. LL, EK, WB, TI, and AH supervised the study. TI and AH obtained funding and material support. All authors critically revised drafts of the manuscript and approved the final manuscript.

## Conflict of Interest

The authors declare that the research was conducted in the absence of any commercial or financial relationships that could be construed as a potential conflict of interest.

## References

[1] Bellentani S Scaglioni F Marino M Bedogni G. Epidemiology of non-alcoholic fatty liver disease. Dig Dis. (2010) 28:155–61. 10.1159/000282080 20460905

[2] Baumeister SE Völzke H Marschall P John U Schmidt CO Flessa S. Impact of fatty liver disease on health care utilization and costs in a general population: a 5-year observation. Gastroenterology. (2008) 134:85–94. 10.1053/j.gastro.2007.10.024 18005961

[3] Nash DM Ivers NM Young J Jaakkimainen RL Garg AX Tu K. Improving care for patients with or at risk for chronic kidney disease using electronic medical record interventions: a pragmatic cluster-randomized trial protocol. Can J Kidney Health Dis. (2017) 4:2054358117699833. 10.1177/2054358117699833 28607686 PMC5453629

[4] Bazick J Donithan M Neuschwander-Tetri BA Kleiner D Brunt EM Wilson L. Clinical model for NASH and advanced fibrosis in adult patients with diabetes and NAFLD: guidelines for referral in NAFLD. Diabetes Care. (2015) 38:1347–55. 10.2337/dc14-1239 25887357 PMC4477334

[5] Lomonaco R Chen J Cusi K. An endocrine perspective of nonalcoholic fatty liver disease (NAFLD). Ther Adv Endocrinol Metab. (2011) 2:211–25. 10.1177/2042018811419157 23148186 PMC3474641

[6] Chalasani N Younossi Z Lavine JE. The diagnosis and management of non-alcoholic fatty liver disease: practice guideline by the American Association for the Study of Liver Diseases, American College of Gastroenterology, and the American Gastroenterological Association. Am J Gastroenterol. (2012) 107:811−26. 10.1038/ajg.2012.128 22641309

[7] Browning JD Szczepaniak LS Dobbins R Nuremberg P Horton JD Cohen JC. Prevalence of hepatic steatosis in an urban population in the United States: impact of ethnicity. Hepatology. (2004) 40:1387–95. 10.1002/hep.20466 15565570

[8] Zhu JZ Dai YN Wang YM Zhou QY Yu CH Li YM. Prevalence of nonalcoholic fatty liver disease and economy. Dig Dis Sci. (2015) 60:3194–202. 10.1007/s10620-015-3728-3 26017679

[9] Liu J Fox CS Hickson D Bidulescu A Carr JJ Taylor HA. Fatty liver, abdominal visceral fat, and cardiometabolic risk factors: the Jackson Heart Study. Arterioscler Thromb Vasc Biol. (2011) 31:2715–22. 10.1161/ATVBAHA.111.234062 21885852 PMC3228266

[10] Guerrero R Vega GL Grundy SM Browning JD. Ethnic differences in hepatic steatosis: an insulin resistance paradox? Hepatology. (2009) 49:791–801. 10.1002/hep.22726 19105205 PMC2675577

[11] Cordero P Campion J Milagro FI Martinez JA. Transcriptomic and epigenetic changes in early liver steatosis associated to obesity: effect of dietary methyl donor supplementation. Mol Genet Metab. (2013) 110:388–95. 10.1016/j.ymgme.2013.08.022 24084163

[12] Cordero P Gomez-Uriz AM Campion J Milagro FI Martinez JA. Dietary supplementation with methyl donors reduces fatty liver and modifies the fatty acid synthase DNA methylation profile in rats fed an obesogenic diet. Genes Nutr. (2013) 8:105–13. 10.1007/s12263-012-0300-z 22648174 PMC3534997

[13] Dai Y Zhu J Meng D Yu C Li Y. Association of homocysteine level with biopsy-proven non-alcoholic fatty liver disease: a meta-analysis. J Clin Biochem Nutr. (2016) 58:76–83. 10.3164/jcbn.15-54 26798201 PMC4706092

[14] Frelut ML Emery-Fillon N Guilland JC Dao HH de Courcy GP. Alanine amino transferase concentrations are linked to folate intakes and methylenetetrahydrofolate reductase polymorphism in obese adolescent girls. J Pediatr Gastroenterol Nutr. (2006) 43:234–9. 10.1097/01.mpg.0000228110.83616.92 16877991

[15] Han JM Jo AN Lee SM Bae HS Jun DW Cho YK. Associations between intakes of individual nutrients or whole food groups and non-alcoholic fatty liver disease among Korean adults. J Gastroenterol Hepatol. (2014) 29:1265–72. 10.1111/jgh.12520 24955455

[16] Koplay M Gulcan E Ozkan F. Association between serum vitamin B12 levels and the degree of steatosis in patients with nonalcoholic fatty liver disease. J Investig Med. (2011) 59:1137–40. 10.2310/JIM.0b013e31822a29f5 21804402

[17] Tryndyak V de Conti A Kobets T Kutanzi K Koturbash I Han T. Interstrain differences in the severity of liver injury induced by a choline- and folate-deficient diet in mice are associated with dysregulation of genes involved in lipid metabolism. FASEB J. (2012) 26:4592–602. 10.1096/fj.12-209569 22872676 PMC3475259

[18] Zolfaghari H Askari G Siassi F Feizi A Sotoudeh G. Intake of nutrients, fiber, and sugar in patients with nonalcoholic fatty liver disease in comparison to healthy individuals. Int J Prev Med. (2016) 7:98. 10.4103/2008-7802.188083 27625763 PMC4995850

[19] Hirsch S Poniachick J Avendaño M Csendes A Burdiles P Smok G. Serum folate and homocysteine levels in obese females with non-alcoholic fatty liver. Nutrition. (2005) 21:137–141. 10.1016/j.nut.2004.03.022 15723740

[20] Vilar L Oliveira CP Faintuch J Mello ES Nogueira MA Santos TE. High-fat diet: a trigger of non-alcoholic steatohepatitis? Preliminary findings in obese subjects. Nutrition. (2008) 24:1097–102. 10.1016/j.nut.2008.05.017 18640006

[21] Yang QH Carter HK Mulinare J Berry RJ Friedman JM Erickson JD. Race-ethnicity differences in folic acid intake in women of childbearing age in the United States after folic acid fortification: findings from the National Health and Nutrition Examination Survey, 2001-2002. Am J Clin Nutr. (2007) 85:1409–16. 10.1093/ajcn/85.5.1409 17490980

[22] Vaccaro JA Huffman FG. Race/ethnicity, gender- and age-specific differences in micronutrient intakes of US adults with and without diabetes. Int J Food Sci Nutr. (2013) 64:175–84. 10.3109/09637486.2012.710894 22856382

[23] Yu D Sonderman J Buchowski MS McLaughlin JK Shu XO Steinwandel M. Healthy eating and risks of total and cause-specific death among low-income populations of african-americans and other adults in the Southeastern United States: A Prospective Cohort Study. PLoS Med. (2015) 12:e100. discussion e1001830. 10.1371/journal.pmed.1001830 26011727 PMC4444091

[24] The SCCS Cohort. Available online at: http://www.southerncommunitystudy.org/cohort-description.html (accessed August 18, 2017).

[25] Signorello LB Hargreaves MK Blot WJ. The Southern Community Cohort Study: investigating health disparities. J Health Care Poor Underserved. (2010) 21(1 Suppl.):26–37. 10.1353/hpu.0.0245 20173283 PMC2940058

[26] Lipworth L Okafor H Mumma MT Edwards TL Roden DM Blot WJ. Race-specific impact of atrial fibrillation risk factors in blacks and whites in the southern community cohort study. Am J Cardiol. (2012) 110:1637–42. 10.1016/j.amjcard.2012.07.032 22922000 PMC3496834

[27] Younossi ZM Otgonsuren M Henry L Venkatesan C Mishra A Erario M. Association of nonalcoholic fatty liver disease (NAFLD) with hepatocellular carcinoma (HCC) in the United States from 2004 to 2009. Hepatology. (2015) 62:1723–30. 10.1002/hep.28123 26274335

[28] Signorello LB Buchowski MS Cai Q Munro HM Hargreaves MK Blot WJ. Biochemical validation of food frequency questionnaire-estimated carotenoid, alpha-tocopherol, and folate intakes among African Americans and non-Hispanic Whites in the Southern Community Cohort Study. Am J Epidemiol. (2010) 171:488–97. 10.1093/aje/kwp402 20061366 PMC2842194

[29] Ainsworth BE Haskell WL Whitt MC Irwin ML Swartz AM Strath SJ. Compendium of physical activities: an update of activity codes and MET intensities. Med Sci Sports Exerc. (2000) 32(9 Suppl.):S498–504. 10.1097/00005768-200009001-00009 10993420

[30] Willett WC Howe GR Kushi LH. Adjustment for total energy intake in epidemiologic studies. Am J Clin Nutr. (1997) 65(4 Suppl.):1220S−8S. discussion 1229S−31S. 10.1093/ajcn/65.4.1220S 9094926

[31] Kiage JN Sampson UK Lipworth L Fazio S Mensah GA Yu Q. Intake of polyunsaturated fat in relation to mortality among statin users and non-users in the Southern Community Cohort Study. Nutr Metab Cardiovasc Dis. (2015) 25:1016–24. 10.1016/j.numecd.2015.07.006 26298428 PMC4637133

[32] Tinker SC Hamner HC Qi YP Crider KS. US women of childbearing age who are at possible increased risk of a neural tube defect-affected pregnancy due to suboptimal red blood cell folate concentrations, National Health and Nutrition Examination Survey 2007 to 2012. Birth Defects Res A Clin Mol Teratol. (2015) 103:517–26. 10.1002/bdra.23378 25884850 PMC4515959

[33] Dowd JB Aiello AE. Did national folic acid fortification reduce socioeconomic and racial disparities in folate status in the US? Int J Epidemiol. (2008) 37:1059–66. 10.1093/ije/dyn066 18456713

[34] Younossi ZM Stepanova M Afendy M Fang Y Younossi Y Mir H. Changes in the prevalence of the most common causes of chronic liver diseases in the United States from 1988 to 2008. Clin Gastroenterol Hepatol. (2011) 9:524–30.e521; quiz e560. 10.1016/j.cgh.2011.03.020 21440669

[35] Meng YY Diamant A Jones J Lin W Chen X Wu SH. Racial and ethnic disparities in diabetes care and impact of vendor-based disease management programs. Diabetes Care. (2016) 39:743–9. 10.2337/dc15-1323 26965718

[36] Ogden CL Carroll MD Fryar CD Flegal KM Prevalence of obesity among adults and youth: United States, 2011-2014. NCHS Data Brief . (2015) 219:1–8. 26633046

[37] Wells JC. Ethnic variability in adiposity, thrifty phenotypes and cardiometabolic risk: addressing the full range of ethnicity, including those of mixed ethnicity. Obes Rev. (2012) 13 (Suppl. 2):14–29. 10.1111/j.1467-789X.2012.01034.x 23107256

[38] Dunn W Sanyal AJ Brunt EM Unalp-Arida A Donohue M McCullough AJ. Modest alcohol consumption is associated with decreased prevalence of steatohepatitis in patients with non-alcoholic fatty liver disease (NAFLD). J Hepatol. (2012) 57:384–91. 10.1016/j.jhep.2012.03.024 22521357 PMC3399018

[39] Anstee QM Day CP. The genetics of NAFLD. Nat Rev Gastroenterol Hepatol. (2013) 10:645–55. 10.1038/nrgastro.2013.182 24061205

