# Supplementary material for: A Description of Risk Factors for Non-alcoholic Fatty Liver Disease in the Southern Community Cohort Study: A Nested Case-Control Study
Source: Front Nutr. 2020 May 21;7:71. doi: 10.3389/fnut.2020.00071 (PMC7326146; doi:10.3389/fnut.2020.00071)
Supplement: Supplementary file 1 [file Table_1.DOCX]

**Supplemental Table 1**

| **Variables** | **Participants who were not included in the study (N = 2371)** | **Percent missing from participants who were not included in the study** |
| --- | --- | --- |
| Age (years) | 56 (49, 62) | 0% |
| Race  Black  White | 1298 (55%)  998 (42%) | 3% |
| Sex  Female  Male | 1791 (76%)  577 (24%) | 0% |
| History of diabetes | 693 (29%) | 6% |
| History of hypercholesterolemia | 955 (40%) | 23% |
| History of MI or bypass | 232 (10%) | 6% |
| BMI (kg/m^2^) | 30.0 (25.5, 36.4) | 10% |
| Household income < $15,000/year | 1425 (60%) | 11% |
| Number of alcoholic drinks/day among current alcohol users | 0.3 (0.07, 2.0) | 13% |
| Current alcohol use | 842 (36%) | 13% |
| Daily energy expenditure (MET-hrs/day) | 12.5 (6.3. 21.8) | 11% |
| Last visit to a doctor (prior to enrollment, in months) | 1 (0, 3) | 16% |
| Daily energy intake (kcal/day) | 1973.3 (1370.8, 2813.3) | 26% |
| Total daily fat intake (g/day) | 74.5 (50.2, 111.0) | 26% |
| Total daily folate intake (μg/day) | 410.6 (280.4 600.9) | 26% |
